# Predictors of chronic prescription opioid use after orthopedic surgery: derivation of a clinical prediction rule.

**DOI:** 10.1186/s13741-018-0105-8

**Published:** 2018-11-22

**Authors:** Daniel I Rhon, Suzanne J Snodgrass, Joshua A Cleland, Charles D Sissel, Chad E Cook

**Affiliations:** 1 Center for the Intrepid, Brooke Army Medical Center, 3551 Roger Brooke Drive, JBSA Fort Sam, Houston, TX 78234 USA; 2 Doctoral Program in Physical Therapy, Baylor University, San Antonio, TX USA; 3 School of Health Sciences, Faculty of Health and Medicine, The University of Newcastle, University Drive, Callaghan, NSW Australia; 4 Department of Physical Therapy, Franklin Pierce University, Manchester, NH USA; 5 Program Analysis and Evaluation Division, US Army Medical Command, Joint Base San Antonio - Fort Sam Houston, San Antonio, TX 78234 USA; 6 Division of Physical Therapy, Department of Orthopedics, Duke University, Duke MSK, Duke Clinical Research Institute, Durham, NC USA

**Keywords:** Prescription opioids, Opioids, Chronic opioid use, Hip surgery, Postoperative care

## Abstract

**Background:**

Prescription opioid use at high doses or over extended periods of time is associated with adverse outcomes, including dependency and abuse. The aim of this study was to identify mediating variables that predict chronic opioid use, defined as three or more prescriptions after orthopedic surgery.

**Methods:**

Individuals were ages between 18 and 50 years and undergoing arthroscopic hip surgery between 2004 and 2013. Two categories of chronic opioid use were calculated based on individuals (1) having three or more unique opioid prescriptions within 2 years and (2) still receiving opioid prescriptions > 1 year after surgery. Univariate elationships were identified for each predictor variable, then significant variables (*P* > 0.15) were entered into a multivariate logistic regression model to identify the most parsimonious group of predictor variables for each chronic opioid use classification. Likelihood ratios were derived from the most robust groups of variables.

**Results:**

There were 1642 participants (mean age 32.5 years, SD 8.2, 54.1% male). Nine predictor variables met the criteria after bivariate analysis for potential inclusion in each multivariate model. Eight variables: socioeconomic status (from enlisted rank family), prior use of opioid medication, prior use of non-opioid pain medication, high health-seeking behavior before surgery, a preoperative diagnosis of insomnia, mental health disorder, or substance abuse were all predictive of chronic opioid use in the final model (seven variables for three or more opioid prescriptions; four variables for opioid use still at 1 year; all< 0.05). Post-test probability of having three or more opioid prescriptions was 93.7% if five of seven variables were present, and the probability of still using opioids after 1 year was 69.6% if three of four variables were present.

**Conclusion:**

A combination of variables significantly predicted chronic opioid use in this cohort. Most of these variables were mediators, indicating that modifying them may be feasible, and the potential focus of interventions to decrease the risk of chronic opioid use, or at minimum better inform opioid prescribing decisions. This clinical prediction rule needs further validation.

## Background

Opioid prescriptions for managing non-cancer-related pain have been on the rise in the US, reaching epidemic proportions (Kolodny et al. 2015). This is problematic as the number of deaths from opioid overdose are also rising, increasing by 27.6% from 2015 to 2016, and 34.5% from 2016 to 2017 in the US (Vivolo-Kantor et al. 2018). Between 21 and 43% of individuals that take prescriptions opioids for chronic musculoskeletal pain will misuse them or develop substance abuse disorders (Ives et al. 2006; Martell et al. 2007; Vowles et al. 2015).

Multiple clinical practice guidelines address opioid prescription for chronic non-cancer pain indicating that opioids should not be considered the first line of treatment (Dowell et al. 2016; Nuckols et al. 2014). However, there is less focused on opioid use for acute pain, such as after traumatic injuries or surgical procedures. For the most part, the latter has been warranted and accepted as standard clinical practice for pain management (Hegmann et al. 2014; Macintyre et al. 2014). However, this practice still merits caution as there is concern some patients may become chronic users after being treated for acute pain (Frieden and Houry 2016; Kaplovitch et al. 2015). As many as 13% of opioid-naive individual undergoing orthopedic surgery may go on to chronic opioid use (Johnson et al. 2016). Orthopedic surgeons are the third highest prescribers of opioid pain medication (Morris and Mir 2015), as they must help their patients adequately manage acute pain during the postoperative period. Dosage patterns of opioid prescriptions have been shown to influence chronic opioid use after orthopedic surgery (Cook et al. 2017; Kim et al. 2017), but there may be other influential variables that help predict chronic use.

For these reasons, identification of risk factors that predict misuse of prescription opioids has been the target of much research (Cochran et al. 2014; Kaye et al. 2017; Skala et al. 2013). However, the majority of research has focused on abuse (such as misuse, addiction, and aberrant behavior) and less on chronic use (proper use over a longer period of time). Chronic opioid use is associated with numerous potential adverse effects (Baldini et al. 2012), many of which develop over time (Els et al. 2017). Information that may help predict whether a patient is at higher risk of becoming a chronic opioid user is vital for informing optimal clinical decision-making, such as identifying which comorbidities associated with chronic use and targeting them for earlier interventions.

The purpose of this study was to identify patient variables that predicted chronic prescription opioid use in the 2 years following arthroscopic hip surgery.

## Methods

### Study design

The study was an observational cohort of patients within the Military Health System (MHS) that underwent arthroscopic hip surgery between 30 June, 2004, and 1 July, 2013.

### Setting

Data were derived from the MHS Data Repository (MDR), which captures and tracks all medical visits for all beneficiaries of the Department of Defense (DoD). This includes retired, active military, and service family members. The MDR is the centralized data repository that captures, archives, validates, integrates, and distributes Defense Health Agency corporate health care data worldwide. Any medical visit, in a military or civilian setting, where the DoD insurance plan TRICARE is the payer (covering 100% of armed services personnel and their dependents) is captured in the MDR.

### Participants

**Fig. 1:**
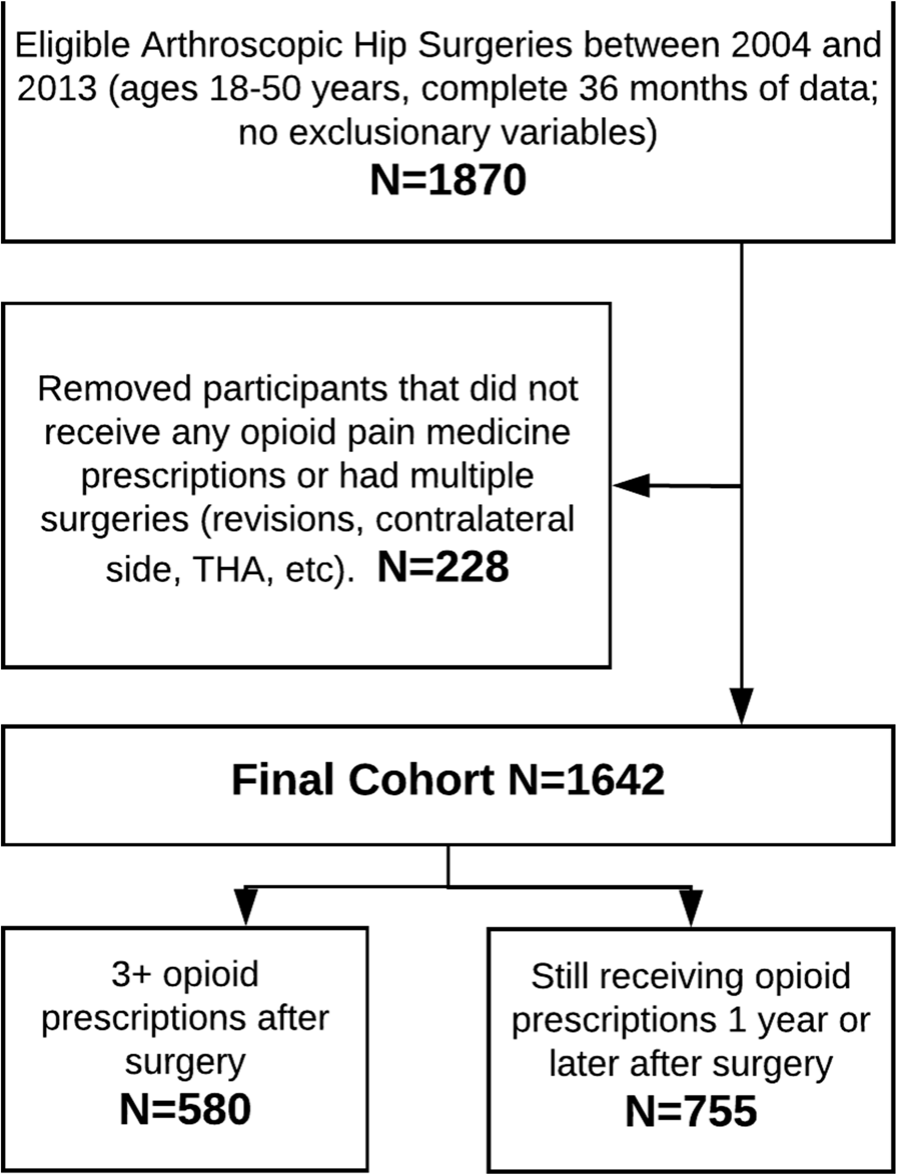
Cohort extraction

To keep the population homogenous, the intent was to identify adult patients undergoing hip arthroscopy specifically for femoroacetabular impingement (FAI) syndrome. FAI syndrome is a musculoskeletal disorder of the hip more common in younger adults and often treated with surgical correction of joint morphology (Amanatullah et al. 2015; Fayad et al. 2013). In fact, it is the most common reason for arthroscopic hip surgery in younger, active adults in civilian as well as military populations (Dutton et al. 2016), with a fivefold increase in the US between 2005 and 2013 (Kremers et al. 2017) Therefore, subjects under 18 or over 50 years of age were excluded, leaving those that best represents the age range for symptomatic FAI syndrome (Clohisy et al. 2013). Because FAI syndrome does not have a diagnosis code established by the International Classification of Diseases (ICD-9), we identified surgical procedures used to treat this condition, in order to make the cohort more homogenous. Any subject with a recorded encounter in the system that specifically included an arthroscopic hip procedure, identified by Current Procedural Terminology (CPT) codes of 29914, 29915, 29916, and 29862, was included in the cohort. All subjects with potential confounding diagnosis codes present prior to the surgery, which could otherwise rationalize the need for arthroscopic hip surgery, were excluded (hip osteoarthritis, hip avascular necrosis, hip or pelvis fracture, or neoplasm). Patients with any additional hip surgeries (revisions, contralateral side, or hip arthroplasty) during the 2-year follow-up period were also excluded. All patients that were not eligible beneficiaries in the DHA health insurance plan for 12 months before and 24 months after surgery were also excluded. Finally, only subjects that received opioid prescriptions after surgery were included in the analyses (Fig. 1). Additional details of the extraction for cohort have been published and available (Rhon et al. 2018).

### Reporting guidelines

The Transparent Reporting of multivariate prediction model for Individual Prognosis Or Diagnosis (TRIPOD) statement for developmental prediction models was used to guide the reporting of this study (Collins et al. 2015). Ethical approval for the study was granted by the Brooke Army Medical Center Institutional Review Board.

### Data sources/measurement

Data from MDR includes person-level data for all outpatient and inpatient medical visits, in both military and civilian hospitals. Within the US, the data from the MDR reflects a single-payer system as compared to the more common private-insurance-based system. It also includes any prescriptions filled, to include total unique prescriptions and days’ supply of medication for each prescription. The data were abstracted and aggregated by a senior healthcare analyst working for the Army Medical Command with over 10 years of experience and who routinely aggregates data of this nature. De-identified data were provided to the investigators in raw form (one line for each unique medical visit) and also in an aggregated file at the single-person level, with a total sum of each care variable for each unique subject. The aggregate data was validated against the raw data by a different investigator (other than the healthcare analyst), and if any questions arose or further clarification was needed, then the issue was brought to the attention of the senior analyst for consensus.

### Study variables

#### Descriptive variables

Patient characteristics included mean age, sex, total healthcare visits (including those unrelated to surgery), and total healthcare costs (including those unrelated to surgery), sex, active duty status, socioeconomic status (categorized as officer or enlisted), and location of surgery (military treatment facility (MTF) or civilian hospital).

#### Outcome variables

Opioid prescriptions were identified by using the American Hospital Formulary Service (AHFS) therapeutic class codes (280808 and 280812) found in the Pharmacy Data Transaction Service (PDTS) section of MDR. The prescription date and type are provided at the person level. Because opioids prescribed at the time of surgery are likely associated with the initial dose provided to manage immediate postoperative pain, occurrences of opioid prescriptions within the immediate perioperative window (0–2 days) were excluded from the frequency counts. Current clinical practice guidelines include prescription of opioid-based medication to manage acute postsurgical pain, and therefore, we expected most patients to have at least one prescription immediately after surgery. However, we were more interested in subsequent prescriptions and management patterns beyond this perioperative prescription. There were two outcomes used in this study. The first was defined by unique individuals with three or more unique opioid prescriptions within a 24-month timeframe (designated as three or more opioids). The second was defined as unique individuals who received any opioid prescriptions that took place at least 1 year or more after surgery (designated as “1 year+” cohort). The first outcome allowed us to assess frequency and has been used to define chronic opioid use in previous studies (DeVries et al. 2014; Yang et al. 2015; Zarling et al. 2016). The second outcome allowed for a more temporal assessment over time. We did not assess perioperative prescription dosing variables at baseline, as the goal was to capture clinical practice delivered in a pragmatic manner, allowing for the individualized variations required in dosing for immediate postoperative pain management.

#### Predictor variables

In recognition that various comorbidities can influence general healthcare utilization, a number of comorbidities were identified based on a medical visit with a corresponding ICD-9 code. A recent systematic review identified nine predictive models for determining opioid abuse, and the majority were based on diagnosis codes (ICD-9) (Alzeer et al. 2018). Specific details for how these variables were extracted and their relevance to prognosis in individuals with musculoskeletal outcomes have been published (Rhon et al. 2018). Seventeen variables were identified as potential predictors. Demographic predictors included age, sex, location of surgery, and socioeconomic status. Military rank was used to define socioeconomic status, as a proxy measure of education, income, and cultural role. Few enlisted personnel (7.6%, 2015 data) have a Bachelor’s degree or higher (Office of the Deputy Assistant Secretary of Defense for Military Community and Family Policy (ODASD (MC&FP)) 2015) compared to nearly 100% of military officers, who usually commence military service with a Bachelor’s degree or are expected to have it within the first few years of service. Incomes are higher for higher ranking military officers, and though it is possible that their spouses have high incomes increasing the family’s socioeconomic status, spouse income is unlikely to influence socioeconomic status more than rank. Even so, spousal income would likely favor higher ranking personnel because less enlisted personnel (51%) are married compared to military officers (69.6%) (Office of the Deputy Assistant Secretary of Defense for Military Community and Family Policy (ODASD (MC&FP)) 2015). Finally, military-assigned housing is often geographically segregated by rank reflecting the military culture, e.g., lower ranked enlisted personnel are typically housed in smaller homes in one geographic area, with senior officers in much larger homes in a different geographic area.

Medical history predictors included preoperative diagnosis of insomnia, mental health disorder, substance abuse, or presence of chronic pain. Behavioral-based predictors included preoperative opioid use (Sing et al. 2016; Zarling et al. 2016; Zywiel et al. 2011), preoperative non-opioid pain medication use, and high health-seeking behavior [defined by dividing the total health visits into quartiles and dichotomizing the groups into low healthcare-seeking (quartile 1 through 3) and high healthcare-seeking (quartile 4)]. Care-oriented predictors included three or more visits of rehabilitation for the hip, occurrence of a hip infection, surgical procedures of femoroplasty (cam lesion), acetabuloplasty (pincer lesion), and arthroscopic repair of labrum. Specific diagnosis and procedure codes used for each category in this cohort have been published (Rhon et al. 2018).

### Statistical approach

Our methodology involves cluster predictive analyses, a form of multivariate predictive modeling that appropriately identifies patterns associated with the predicted variable. Cluster predictive analyses, sometimes referred to as clinical prediction rules, are especially beneficial when the model incorporates standard patient-level or clinical-level factors that are readily available in most clinician-patient encounters.

All analyses were performed using SPSS version 24.0 (IBM Corp. Armonk, NY, USA). Descriptive statistics representing raw data for the categories of three or more prescriptions of opioids and < 3 prescriptions of opioids were calculated, including means, standard deviations, and frequencies and distributions, where appropriate. Bivariate assessments were provided to determine differences across groups.

Bivariate relationships were analyzed with 17 individual logistic regression analyses for both outcome variables [(1) three or more unique prescriptions and (2) still receiving prescriptions at 1 year or greater]. For each analysis, odds ratios and 95% confidence intervals were captured, as well as *p* values and Nagelkerke R^2^ measures. A Nagelkerke R^2^ is a goodness of fit measure that reflects the explanatory strength of the predictor within a model (Bewick et al. 2005). Values closer to 1.0 suggest strong explanation whereas values near zero suggest only weak explanation.

The univariate findings from the bivariate logistic regression analyses for both outcome measures (three or more prescriptions of opioids and opioid prescription of 1 year or greater) that exhibited *p* values of < 0.05 were retained for the multivariate regression analysis. To assure appropriate modeling, a multicollinearity assessment for each of the retained variables was performed using correlation matrices. A correlational finding of *r* > 0.7 between independent variables was used to assess the potential of multicollinearity (Shen and Gao 2008). Since no variables exhibited a correlation greater than 0.4, all variables were retained for both multivariate models. Because there is some overlap with variables, we chose to adjust only for military status (active duty service member or other, to include family member or retired service member) and socioeconomic status (four categories: junior or senior enlisted and junior or senior officer), as these are best supported in the literature (Bennett et al. 2013; Edlund et al. 2014) and the cohort was relatively homogenous already with no influence on dependent variables found through independent analysis of other factors.

For the multivariate analyses, a backward stepwise logistic regression was used. For both multivariate models, a *p* value of ≤ 0.05 was considered significant for the bivariate analyses, whereas 95% confidence intervals that did not cross 1 were considered significant for all likelihood ratio analyses. Variables retained by the regression model were used to create conditions, a unique feature of a clinical prediction rule (CPR). Depending on the number of variables retained in the stepwise regression, findings were inputted into 2 × 2 contingency tables that involved the conditions of 1 of *X*, 2 of *X*, 3 of *X*, and so on. For each condition, sensitivity, specificity, and likelihood ratios and 95% CIs were calculated. In each condition, post-test probability measures were calculated using pretest probabilities within the sample. For the first multivariate model, the pre-test probability of three or more opioids prescriptions was 35.5% whereas the pre-test probability of an opioid prescription of 1 year or longer was 53.1%. We calculated post-test probability of a negative and positive finding using a post-test probability calculator.

## Results

There were 1642 individuals that met the criteria and were included in analysis. There were notable differences among those who received three or more opioid prescriptions and those who did not, including age, sex socioeconomic status, and healthcare utilization. A greater rate of individuals in the three or more opioid prescriptions utilized prescription opioids prior to surgery (50.7% vs 34.7%). Higher medical costs and visits (both general and specifically hip related) were present in the three or more opioid prescription group as well (Table 1). The mean total days’ supply of opioids was much higher in the three or more opioid prescription group (125.7 days vs 5.7 days).

**Table 1:** Descriptive statistics (*N*, %) for the total sample and those with and without three or more opioid prescriptions (*p* value compares these two groups)

Variable	Total sample (*N* = 1642)	Individuals with 3 or more prescriptions for opioids (*N* = 580)	Individuals with less than 3 or no prescriptions for opioids (*N* = 1062)	*P* value	Individuals still receiving opioid prescriptions after 1 year (*N* = 755)	Individuals not receiving opioid prescriptions after 1 year (*N* = 887)	*P* value
Age (SD)	32.45 (8.18)	31.37 (7.74)	32.85 (8.39)	< 0.01*	32.9 (8.5)	31.9 (7.8)	< 0.01*
Sex—female	754 (45.9)	298 (51.4)	456 (42.9)	< 0.01*	388 (51.4)	366 (41.3)	< 0.01*
Active duty family (service or family member	1108 (67.5)	387 (66.7)	721 (67.9)	0.63	523 (69.3)	585 (66.0)	0.15
Service							
Army	783 (47.7)	290 (50.0)	435 (41.0)	< 0.01*	351 (46.5)	374 (42.3)	0.29
Coast Guard	29 (1.8)	7 (1.2)	22 (2.1)		11 (1.5)	18 (2.0)	
Air Force	448 (27.3)	134 (23.1)	280 (26.4)		187 (24.8)	227 (25.6)	
Marine Corps	214 (13.0)	65 (11.2)	129 (12.1)		83 (11.0)	111 (12.5)	
Navy	288 (17.5)	84 (14.5)	187 (17.6)		122 (16.2)	149 (16.8)	
Other	8 (0.5)	0	7 (0.5)		1	6 (0.5)	
Socioeconomic status				< 0.01*			< 0.01*
Junior enlisted	390 (23.8)	182 (31.4)	208 (19.6)		202 (26.8)	188 (21.2)	
Senior enlisted	842 (51.3)	305 (52.6)	537 (50.7)		418 (55.4)	424 (47.8)	
Junior officer	200 (12.2)	54 (9.3)	146 (13.7)		71 (9.4)	129 (14.5)	
Senior officer	192 (11.7)	39 (6.7)	153 (14.4)		64 (8.5)	128 (14.4)	
Unknown	18 (1.1)	0	18 (1.7)		0	18 (2.0)	
Military hospital [versus civilian]	837 (51.0)	293 (50.5)	544 (51.2)	0.78	373 (49.4)	464 (52.3)	0.24
Total medical care within 2 years after surgery (SD)							
Mean medical visits	80.94 (67.13)	119.58 (86.50)	59.83 (40.31)	< 0.01*	105.1 (80.0)	60.4 (44.6)	< 0.01*
Mean medical costs	$25,380 ($24,604)	$36,199 ($32,372)	$19,471 ($16,292)	< 0.01*	$32,140 ($28,778)	$19,626 ($18,555)	< 0.01*
Mean hip-related medical visits	24.41 (22.31)	32.42 (28.31)	20.04 (17.56)	< 0.01*	29.0 (25.7)	20.5 (18.1)	< 0.01*
Mean hip-related medical costs	$13,185 ($14,187)	$15,665 ($16,119)	$11,831 ($12,819)	< 0.01*	$14,546 ($15,639)	$12,027 ($12,717)	< 0.01*
Individuals with a prescription prior to surgery	662 (40.3)	294 (50.7)	368 (34.7)	< 0.01*	399 (52.8)	416 (46.9)	0.02*
Opioid prescription use after (SD)							
Mean unique prescriptions	5.0 (9.1)	10.3 (12.8)	1.5 (0.9)	< 0.01*	8.4 (12.1)	1.6 (18.1)	< 0.01*
Median unique prescriptions	2.0	6.0	1.0	< 0.01*	5.0	1.0	< 0.01*
Mean total days’ supply	43.0 (137.1)	93.7 (208.2)	10.1 (12.2)	< 0.01*	76.3 (189.8)	10.3 (18.1)	< 0.01*
Median total days’ supply	10.0	35.0	7.0	< 0.01*	23.0	5.0	< 0.01*

*N* (%) unless otherwise indicated

*SD* standard deviation, *MTF* military treatment facility, *Network* non-military medical facility, *Significant *P* < 0.05

Bivariate logistic regression analyses identified eight variables that were significantly associated with receiving three or more opioid prescriptions in a 24-month period (Table 2). Female sex, history of preoperative opioid prescriptions, having received non-opioid-based pain medication prescriptions prior to surgery, high health-seeking behavior, and a preoperative diagnosis of insomnia, chronic pain, substance abuse disorder, or mental health disorder were all associated with higher odds of receiving three or more opioid prescriptions within a 24-month period after surgery.

**Table 2:** Univariate relationships between predictor variables and having three or more opioid prescriptions in the 24-month period after hip surgery, adjusted for socioeconomic and active duty status

Variable	Odds ratio (95% CI)	*P* value	Nagelkerke R^2^
Age	1.01 (0.99, 1.02)	0.44	0.05
Preoperative use of prescription opioids	1.81 (1.46, 2.23)	< 0.01*	0.07
Socioeconomic status (enlisted rank)	1.12 (0.72, 1.73)	0.62	0.05
Sex (female)	1.37 (1.12, 1.69)	< 0.01*	0.06
MTF location for surgery (vs network)	1.07 (0.87, 1.32)	0.49	0.05
Insomnia preoperatively	2.56 (1.79, 3.66)	< 0.01*	0.07
Mental health disorder preoperatively	1.95 (1.51, 2.51)	< 0.01*	0.07
Substance abuse preoperatively	1.68 (1.27, 2.23)	< 0.01*	0.06
Chronic pain diagnosis preoperatively	1.53 (1.08, 2.15)	0.01*	0.05
Non-opioid-based pain medication prescription preoperatively	1.68 (1.30, 2.17)	< 0.01*	0.06
Health-seeking behavior preoperatively	8.38 (5.80, 12.11)	< 0.01*	0.17
At least 3+ visits of hip-related physical therapy preoperatively (based on median)	1.04 (0.84, 1.28)	0.71	0.005
Occurrence of hip infection after	0.50 (0.70, 3.61)	0.49	0.05
Femoroplasty (cam lesion)	0.94 (0.80, 1.09)	0.41	0.05
Acetabuloplasty (pincer lesion)	1.01 (0.82, 1.23)	0.96	0.05
Labral repair	0.93 (0.79, 1.09)	0.38	0.05

*MTF* military treatment facility, *Network* non-military medical facility, * Significant P<0.05

Bivariate logistic regression analyses identified five variables that were significantly associated with ongoing opioid prescriptions beyond 1 year (Table 3). Female sex, lower socioeconomic status, high health-seeking behavior, and a preoperative diagnosis of a substance abuse or mental health disorder were associated with higher odds of receiving a new prescription for opioids 1 year or later after surgery.

**Table 3:** Univariate relationships between predictor variables and still receiving an opioid prescription 1 year or more after hip surgery, adjusted for socioeconomic and active duty status

Variable	Odds ratio (95% CI)	*P* value	Nagelkerke R^2^
Age	0.99 (0.98, 1.01)	0.88	0.02
Preoperative use of prescription opioids	1.19 (0.96, 1.47)	0.11	0.02
Socioeconomic status (enlisted rank)	0.56 (0.35, 0.89)	0.02*	0.02
Sex (female)	1.54 (1.24, 1.91)	< 0.01*	0.03
MTF location for surgery (vs network)	1.13 (0.92, 1.40	0.23	0.02
Insomnia preoperatively	1.23 (0.85, 1.78)	0.27	0.02
Mental health disorder preoperatively	1.45 (1.11, 1.89)	< 0.01*	0.02
Substance abuse preoperatively	1.92 91.48, 2.49)	< 0.01*	0.04
Chronic pain diagnosis preoperatively	1.41 (0.98, 2.03)	0.06	0.02
Non-opioid-based pain medication prescription preoperatively	0.97 (0.75, 1.27)	0.83	0.02
Health-seeking behavior preoperatively	4.48 (3.05, 6.59)	< 0.01*	0.08
At least 3+ visits of hip-related physical therapy preoperatively (based on median)	1.15 (0.93, 1.42)	0.19	0.02
Occurrence of infection after	1.09 (0.15, 7.87)	0.93	0.02
Femoroplasty (cam lesion)	9.95 (0.81, 1.10)	0.50	0.02
Acetabuloplasty (pincer lesion)	0.98 (0.81, 1.21)	0.90	0.02
Labral repair	0.90 (0.77, 1.06)	0.20	0.02

*MTF* military treatment facility, *Network* non-military medical facility, * Significant *P* < 0.05

Multivariate analyses identified seven variables that were associated with receiving three or more opioid prescriptions 24 months after surgery (Table 4). Preoperative prescription opioid use (OR 2.62; 95% CI 2.02, 3.39), preoperative non-opioid pain medication prescription (OR 1.37; 95% CI 1.03, 1.81), high health-seeking behavior (OR 7.23; 95% CI 4.94, 10.54), female sex (OR 1.28; 95% CI 1.02, 1.61), preoperative insomnia (OR 2.09; 95% CI 1.42, 3.09), mental health disorder (OR 2.24; 95% CI 1.61, 3.09), and substance abuse disorder diagnoses (OR 1.45; 95% CI 1.07, 1.98) all contributed to higher odds of receiving three or more opioids in a 24-month period. The Nagelkerke R^2^ was 0.19.

**Table 4:** Results of multivariate analysis demonstrating variables that predict having three or more opioid prescriptions in the 24-month period after hip surgery, adjusted for socioeconomic and active duty status

Variable	Odds ratio (95% CI)	*P* value
Preoperative use of prescription opioids	2.62 (2.02, 3.39)	< 0.01*
Insomnia, preoperatively	2.09 (1.42, 3.09)	< 0.01*
High health-seeking behavior preoperatively	7.23 (4.94, 10.54)	< 0.01*
Substance abuse preoperatively	1.45 (1.07, 1.98)	0.02*
Non-opioid-based pain medication prescription preoperatively	1.37 (1.03, 1.81)	0.03*
Mental health disorder diagnosis preoperatively	2.24 (1.61, 3.09)	< 0.01*
Sex (female)	1.28 (1.02, 1.61)	0.03*

*Significant *P* < 0.05

Multivariate analyses for individuals receiving opioids beyond 1 year postoperatively in a 24-month period identified four variables associated with this outcome (Table 5). Female sex (OR 1.62; 95% CI 1.30, 2.01), preoperative substance abuse disorder (OR 1.50; 95% CI 1.11, 2.04), and high health-seeking behavior (OR 4.39; 95% CI 2.97, 6.47) were associated with higher odds of receiving an opioid prescription at 1 year or later. Being an officer or in an officer family was associated with lower odds of having an opioid prescription 1 year or more after surgery (OR 0.59; 95% CI 0.46, 0.77). The Nagelkerke R^2^ was 0.25.

**Table 5:** Results of multivariate analysis demonstrating variables that predict still receiving an opioid prescription 1 year or more after hip surgery, adjusted for socioeconomic and active duty status

Variable	Odds ratio (95% CI)	*P* value
Socioeconomic status (officer rank)	0.59 (0.46, 0.77)	< 0.01*
Sex (female)	1.62 (1.30, 2.01)	< 0.01*
Substance abuse preoperatively	1.50 (1.11, 2.04)	< 0.01*
Health-seeking behavior preoperatively	4.39 (2.97, 6.47)	< 0.01*

*Significant *P* < 0.05

Table 6 outlines the sensitivity, specificity, and positive and negative likelihood ratios of the clustered models for each outcome variable. In addition, a post-test probability of meeting the selected conditions (e.g., 1 of *X*, 2 of *X*) is provided based on the prevalence of those with three or more opioid prescriptions or those who received opioid prescriptions within the 24 months beyond 1-year post-surgery. As expected, the positive likelihood ratio increases when greater numbers of positive findings in selected conditions are met (e.g., 5 of *X*, 6 of *X*) with decreasing sensitivity of the models. Post-test probabilities of having three or more opioid prescriptions start at 39.9% with at least one variable and rise to 100.0% if at least six or seven of the seven variables are present. Post-test probabilities of having still receiving an opioid prescription 1 year or later after surgery start at 53.3% with at least one variable and rise to 77.7% if all four variables are present (Table 6).

**Table 6:**
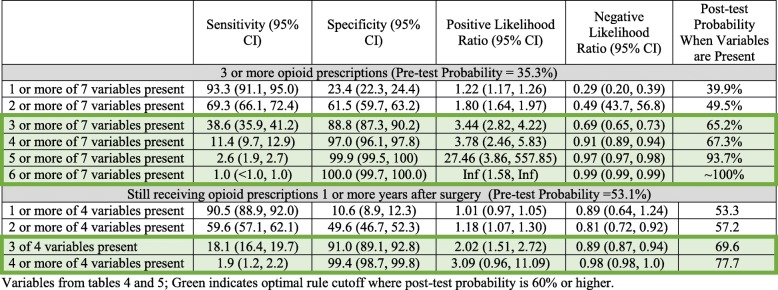
Clinical prediction rule for each of the two chronic opioid utilization definitions

Variables from Tables 4 and 5; green indicates optimal rule cutoff where post-test probability is 60% or higher

## Discussion

The aim of this study was to derive a CPR to identify patients who might be more likely to become chronic users of prescription opioids after orthopedic hip surgery. While other studies have utilized prescription data to define chronic opioid use (Fritz et al. 2018; Sites et al. 2018; Thackeray et al. 2017), this study is the first to develop a clinical prediction rule based on opioid prescription patterns validated from pharmacy data in claims records. The focus of our investigation was to better understand variables that may improve clinical decision-making related to managing patients that are taking opioids after surgery and at the same time generate hypotheses for future trials. Several clinically relevant patient-level and healthcare services utilization variables were identified and include use of non-opioid pain medication prior to surgery, younger age, female, lower socioeconomic status (military rank, representing education and household income), high health-seeking behavior, and presence of substance abuse, insomnia, or mental health disorders prior to surgery. The variables in this CPR were able to identify individuals that received three or more opioid prescriptions in a 2-year period, as well as individuals still receiving new opioid prescriptions at least 1 year after surgery. This CPR can help clinicians identify patients that may be at higher risk become chronic opioid users after orthopedic surgery.

Prior opioid use is one of the strongest predictors of chronic opioid use and poor outcomes (i.e., longer hospital stays, higher rates of pain management referrals, higher rates of postoperative complications) after orthopedic surgery (Chan et al. 2017; Sing et al. 2016), and while it was significant in predicting three or more opioid prescriptions, it was not significant in predicting which individuals were still receiving opioid prescriptions beyond 1 year in our study. It is possible that prior opioid use is related to higher numbers of unique prescriptions, but ones that occur in a shorter period of time. In addition, the presence of a substance abuse disorder diagnoses prior to surgery did predict use beyond 1 year. A diagnosis documented in a medical record may indicate a more substantial dependency problem than the utilization of opioid prescriptions alone. Interestingly, non-opioid pain medication use (most often non-steroidal anti-inflammatory drugs—NSAIDs) before surgery was a significant predictor of chronic opioid use. It may be that for chronic and/or persistent symptoms, patients that had already tried non-opioid-based pain medications before surgery were more likely to make the jump to stronger pain medication after surgery. It is unclear if medical providers considered the preoperative utilization of non-opioid analgesics as satisfying an initial tier of pain management and were more willing to prescribe stronger medication post-surgically. Studies show that prescription patterns for stronger pain medication are dependent on prior history of non-opioid analgesic medication (Muller et al. 2012; Ndlovu et al. 2014).

Females may be at greater risk for chronic opioid use after surgery (Johnson et al. 2016; Walid et al. 2007) and more prone to opioid dependency in general (Back et al. 2011; Unger et al. 2010). Females on higher doses are much less likely to taper down to a lower dose than males (Weimer et al. 2016). While the proportion of females is much lower than males in the military, the rate of injuries is much higher in females (Kodesh et al. 2015; Kucera et al. 2016; Roy et al. 2015). A much higher percent of females in the military are undergoing arthroscopic hip surgery and are much less likely to remain in the military after surgery compared to males (Thomas et al. 2017). In 2015, females made up 16.8% of the military force (Office of the Deputy Assistant Secretary of Defense for Military Community and Family Policy (ODASD (MC&FP)) 2015), but they represent 30% or greater of patients in published hip arthroscopy cohorts in the military (Byrd et al. 2016; Dutton et al. 2016; Thomas et al. 2017). As female sex was as a predictor in our final model, this may be a subset of the population that merits further research when it comes to postoperative pain management.

Socioeconomic status has also been shown to be a risk factor for chronic opioid use (Schoenfeld et al. 2017). Enlisted (Bennett et al. 2013) and younger service members (Ramirez et al. 2017) are much more likely to misuse opioids than officers and older service members. This was corroborated in our findings as well, which identified belonging to an enlisted family was a significant predictor of receiving a new opioid prescription 1 year or later after surgery. These variables should be considered by providers in the Military Health System when crafting pain medication management strategies after surgery.

Orthopedic surgeries are some of the most traumatic, often involving reconstruction of the bone, tendon, and muscle. As such, orthopedic surgeons often prescribe opioids to help manage acute postoperative pain (Morris and Mir 2015). In many cases, this may serve as an initial introduction to opioid medication for a patient. Understanding which variables from a patient’s profile or medical history might lead to a higher risk of chronic opioid use has been identified as a critical need for orthopedic surgeons (Kee et al. 2016). In high-risk cases, perhaps alternate pain management strategies (i.e., non-opioid analgesics) (Martinez et al. 2017; White 2002) could be employed earlier, especially as some of these may be just as effective as opioid-based pain medications, and in some cases superior (Martinez et al. 2017).

Finally, complication rates could potentially influence chronic opioid use. Studies have linked chronic opioid use with higher surgery-related complication rates within the first 90 days following surgery (Sing et al. 2016). Higher levels of opioid prescriptions are associated with greater gastrointestinal complications and longer hospital stays in patients undergoing joint arthroplasty (Mörwald et al. 2018). In our cohort, we excluded anyone with additional hip surgeries (revisions, contralateral surgery, joint arthroplasty), and we accounted for infection, which is one of the most common complications. Therefore, it is more likely that our findings were not affected by these factors. Hip arthroscopy is typically an ambulatory surgical procedure (e.g., same day surgery), so hospital stay would not be a factor in most cases. However, this variable, in addition to any other functional measures (e.g., time to return to walk, time to return to work, time to return to independent activities of daily living) were not available for this study.

### Limitations and future research

It should be noted observational data was used, and therefore, causality cannot be implied. Further, we acknowledge that the results of any observational research rely heavily on the interpretation of the researchers and can be influenced by confounders beyond statistical adjustment. Claims data is limited by the accuracy to which it is entered into the electronic medical records. The opioid utilization data was based on prescriptions, and it is not possible to confirm that patients actually utilized their full prescriptions. However, while this may be the case for those with only one prescription, it is not likely for those that had multiple prescriptions as they would have likely completed one before requesting more. We also do not know the specific reason patients used opioids in a long term as they could have been prescribed for other reasons. Self-report variables were lacking, but would have provided valuable insight (Goesling et al. 2016). Currently, no consensus on a definition for chronic opioid use exists, and several definitions for chronic opioid use have been proposed. Our model is likely to vary based on the definition, as was shown in the two models presented in this study. The stark difference in days’ supply between both the three or more or less than three opioid prescriptions (mean 93.7 vs 10.1 days) and in those still using opioids after 1 year compared to less than 1 year (mean 76.3 vs 10.3 days) indicates that our definition is likely a good proxy for chronic use. There may also be other variables with greater predictive validity, to include self-report measures, complications, and surgical procedures, that were not captured in the current study but may improve prediction algorithms. It was not possible to accurately determine the reasons for the opioid prescriptions, so it is possible that prescriptions were filled for other diagnoses. However, even with a diagnosis linked to the prescription, it would be unknown if the opioids were also acting on the hip pain because of its systemic effects. Finally, this was a specific cohort in a military setting undergoing a surgery to the hip. It is unknown if these findings could be generalized to other populations and for other conditions or body regions. Future prospective studies are needed to better identify relevant variables associated with risk of chronic opioid use after orthopedic surgery.

## Conclusion

In summary, patient variables and medical history may prove informative for understanding the risk of chronic prescription opioid use after surgery. The use of pain medication prior to surgery, younger age, female, lower socioeconomic status (education and household income), high health-seeking behavior, and presence of substance abuse, insomnia, or mental health disorders prior to surgery were all significant in predicting chronic opioid use after surgery. While the presence of a single variable may be helpful, a combination of variables may have greater predictive value for determining the likelihood of chronic opioid use after surgery. As with any initial derivation of a clinical prediction rule, these results need further independent validation in other settings to determine if predictors are consistent.

## Abbreviations

AHFS: American Hospital Formulary Service
CPR: Clinical Prediction Rule
CPT: Current Procedural Terminology
DHA: Defense Health Agency
FAI: Femoroacetabular Impingement
ICD: International Classification of Diseases
MDR: Military Health System Data Repository
NSAID: Non-steroidal anti-inflammatory drug
OR: Odds ratio
PDTS: Pharmacy Data Transaction Service
SD: Standard deviation
SPSS: Statistical Package for Social Sciences
US: United States
